# Hepatic hypertrophy and hemodynamics of portal venous flow after percutaneous transhepatic portal embolization

**DOI:** 10.1186/s12893-019-0486-8

**Published:** 2019-02-18

**Authors:** Shingo Shimada, Toshiya Kamiyama, Hideki Yokoo, Tatsuya Orimo, Kenji Wakayama, Akihisa Nagatsu, Tatsuhiko Kakisaka, Hirofumi Kamachi, Daisuke Abo, Yusuke Sakuhara, Akinobu Taketomi

**Affiliations:** 1 Department of Gastroenterological Surgery I, Hokkaido University Graduate School of Medicine, Kita15-Nishi7, Kita-Ku, Sapporo, Hokkaido 060-8638 Japan; 2 Department of Surgery, Sapporo Kousei Hospital, Kita3-Higashi8, Chuo-Ku, Sapporo, Hokkaido 060-0033 Japan; 3 Department of Radiology, Hokkaido University Graduate School of Medicine, Kita15-Nishi7, Kita-Ku, Sapporo, Hokkaido 060-8638 Japan

**Keywords:** Portal venous flow, Hepatic hypertrophy, Percutaneous transhepatic portal embolization

## Abstract

**Background:**

Percutaneous transhepatic portal embolization (PTPE) is useful for safe major hepatectomy. This study investigated the correlation between hepatic hypertrophy and hemodynamics of portal venous flow by ultrasound sonography after PTPE.

**Methods:**

We analyzed 58 patients with PTPE, excluding those who underwent recanalization (*n* = 10). Using CT volumetry results 2 weeks after PTPE, the patients were stratified into a considerable hypertrophy group (CH; *n* = 15) with an increase rate of remnant liver volume (IR-RLV) ≥ 40% and a minimal hypertrophy group (MH; *n* = 33) with an IR-RLV < 40%. We investigated the hemodynamics of portal venous flow after PTPE and the favorable factors for hepatic hypertrophy.

**Results:**

Univariate and multivariate analysis identified the indocyanine green retention rate at 15 min (ICGR15) and increase rate of portal venous flow volume (IR-pFV) at the non-embolized lobe on day 3 after PTPE as independent favorable factors of IR-RLV. Patients with IR-pFV on day 3 after PTPE ≥100% and ICGR15 ≤ 15% (*n* = 13) exhibited significantly increased IR-RLV compared with others (*n* = 35).

**Conclusions:**

Cases with high IR-pFV on day 3 after PTPE exhibited better hepatic hypertrophy. Preserved liver function and increased portal venous flow on day 3 were important.

## Background

Complete resection of hepatobiliary malignant tumors is the best method to achieve long-term survival [1, 2]. Major hepatectomy is often required for complete resection, and one of the main causes of unresectability is insufficient remnant liver volume (RLV). Percutaneous transhepatic portal embolization (PTPE) is a useful method for safe major hepatectomy for hepatobiliary malignant tumors [3]. Although PTPE promotes hypertrophy in the non-embolized liver and increases the future remnant liver function and volume [4], some patients exhibit insufficient hypertrophy at the non-embolized liver with between 2.8 and 4.5% of patients unable to undergo surgery after PTPE due to insufficient hypertrophy [5, 6]. Furthermore, the degree of hypertrophy and growth rate of the remnant liver after portal vein embolization (PVE) are favorable predictors of post-hepatectomy liver failure [7]. Thus, increased hypertrophy after PTPE is a crucial factor.

Although it was previously reported that hepatic injury (fibrosis or cirrhosis) was an unfavorable factor for hepatic hypertrophy after PTPE [3], favorable factors for hypertrophy after PTPE remain elusive. In addition, the hemodynamics of portal venous flow after PTPE has been not investigated in detail. In this study, we examined the favorable factors for remnant liver hypertrophy and hemodynamics of portal venous flow volume (pFV) after PTPE at the non-embolized lobe by ultrasound sonography (US).

## Methods

### Patients

Between January 2004 and November 2015, 58 patients underwent PTPE for hepatectomy at the Gastroenterological Surgery I unit of Hokkaido University Hospital in Sapporo, Japan. The entire liver volume, liver resection volume, and tumor volume were calculated from contrast-enhanced computed tomography (CT) data by 3D workstations (Virtual Place Lexus; Medical Imaging Laboratory, AZE, Tokyo, Japan, and Synapse Vincent, Fujifilm Medical Co., Ltd., Tokyo, Japan). PTPE was performed in patients with an effective liver resection rate > 60% according to the formula [(liver resection volume – tumor volume) / (whole liver volume – tumor volume)] × 100 [8]. The diagnoses and surgical data of the patients are presented in Table 1. We performed bile duct drainage (mainly nasobiliary drainage) before in cases which T-bil ≥ 2.0 mg/dL. PTPE were performed for the cases which T-bil < 2.0 mg/dL.

**Table 1:** Patient diagnoses and operative procedures

	n
Diagnosis	
Hepatocellular carcinoma	13
Intrahepatic cholangiocarcinoma	4
Bile duct cancer	34
Metastatic liver cancer	2
Gallbladder cancer	5
Operative procedure	
Right hepatectomy	38
Extended right hepatectomy	6
Right trisegmentectomy	3
Left hepatectomy	1
Left trisegmentectomy	4
Right hepatectomy with pancreatoduodenectomy	3
Unresectable cases	3

### PTPE

The PTPE method was previously reported [9] and is briefly described here. Most patients underwent an ipsilateral approach, but the contralateral approach was used when the ipsilateral approach was judged to be unsuitable by interventional radiologists. After the administration of local anesthesia, the intrahepatic portal vein was punctured with an 18-gauge needle (Needle for Ultrasonically Guided Puncture; Create Medic Co., Yokohama, Japan) under US guidance. A 5.5-French sheath introducer (Introducer Set; Medikit Co., Tokyo, Japan) was inserted into the portal vein with a guidewire. Direct portography was performed to evaluate the anatomy and measurement of portal venous pressure (PVP) directly. Next, selective portography was performed with a balloon occlusion catheter (Selecon MP Catheter II; Terumo Co., Tokyo, Japan). The embolic material was absolute ethanol. The method of ethanol injection was previously reported.^9^ After repeat embolization until the resolution of hepatic parenchymal enhancement, direct portography was performed to confirm the PTPE result. Then, we directly measured PVP at the non-embolized lobe. Finally, the 5.5-French sheath was extracted by packing the puncture tract with a gelatin sponge torpedo (Spongel; Astellas Pharma Co., Tokyo, Japan).

### Assessments

The patients’ liver volumes and RLVs were semiautomatically measured by 3D workstations using contrast-enhanced CT imaging data before PTPE and 1 and 2 weeks after PTPE. pFV was measured thrice by pulsed wave Doppler US (Toshiba SSA-700A (Aplio50), Toshiba Medical Systems Co., Ltd., Tokyo, Japan, and LOGIQ P6 BT11, GE Healthcare JAPAN Co., Ltd., Tokyo, Japan) on days 1, 3, 5, and 7 after PTPE. We calculated pFV as following, pFV = portal velocity (pulsed wave Doppler US) × πr (r = radius of portal vein)^2^. We averaged the 3 readings on each day to calculate the pFV. We measured the pFV of the umbilical portion for right-side hepatectomy, the anterior branch for left hepatectomy, and the posterior branch for left trisegmentectomy.

Patients were stratified into a considerable hypertrophy group (CH; *n* = 15) and a minimal hypertrophy group (MH; *n* = 33) based on the increase rate of RLV (IR-RLV), i.e., ≥ 40% versus < 40%, respectively, according to the formula [(RLV at 2 weeks after PTPE – RLV before PTPE) / RLV before PTPE] × 100. We investigated the favorable factors for hepatic hypertrophy and hemodynamics of pFV after PTPE. Using the formula [(pFV after PTPE – pFV before PTPE) / pFV before PTPE] × 100, we evaluated the increase rate of pFV (IR-pFV) at the non-embolized lobe on days 1, 3, 5, and 7 after PTPE by US; the portal venous pressure (PVP) before and after PTPE; patients’ age and sex; hemoglobin, white blood cell, C-reactive protein, aspartate aminotransferase, alanine aminotransferase, platelet, cholinesterase, albumin, total bilirubin (T-bil), and A1c glycated hemoglobin levels; hepatitis B surface antigen; hepatitis C virus antibody; the indocyanine green retention rate at 15 min (ICGR15); pathological liver fibrosis; and the receptor index [uptake ratio of the liver to that of the liver plus heart at 15 min of technetium 99 m diethylenetriaminepentaacetic acid-galactosyl-human serum albumin scintigraphy (LHL15)]. Patients who had portal venous recanalization after PTPE were excluded (*n* = 10, 17.2%).

We performed the assessment of hypertrophy by CT at 2 weeks because most cases (86%, 50/58) underwent hepatectomy with satisfaction for our criteria at 2 weeks after PTPE.

This study was approved by the Hokkaido University Hospital Voluntary Clinical Study Committee and was performed according to Helsinki Declaration guidelines.

### Statistical analysis

Values are expressed as the mean ± standard deviation**.** Univariate analyses were performed using Student’s *t*-test for continuous variables and the chi-square test for non-continuous variables. Multivariate analyses were performed using logistic regression model analyses. Pearson’s correlation coefficients were used to analyze the correlation between the IR-pFV at the non-embolized lobe by US and the IR-RLV. A *P*-value of < 0.05 was considered significant. Statistical analyses were performed using JMP Pro 12.0.1 for Windows (SAS Institute Inc., NC, USA).

## Results

### Perioperative data of patients

Patient perioperative data are provided in Table 1. The diagnoses in our study cohort were hepatocellular carcinoma (*n* = 13), intrahepatic cholangiocarcinoma (*n* = 4), bile duct cancer (*n* = 34), metastatic liver cancer (*n* = 2), and gallbladder cancer (*n* = 5). The operative procedures were right hepatectomy (*n* = 38), extended right hepatectomy (*n* = 6), right trisegmentectomy (*n* = 3), left hepatectomy (*n* = 1), left trisegmentectomy (*n* = 4), and right hepatectomy with pancreatoduodenectomy (*n* = 3). Three tumors were unresectable (*n* = 3). The mean waiting time from PTPE to surgery was 26 days (15–96). Table 2 reports total hepatic volume, RLV, effective liver resection rate before and after PTPE. The mean RLVs before and after PTPE were 438.6 ± 106 mL and 558.8 ± 99.9 mL, respectively (*P* < 0.01). The median IR-RLV was 30.5%. Only one complication occurred, namely, hemobilia (1.7%). No patient exhibited transient thrombocytopenia (defined as a platelet count less than 50 × 10^3^ cells/μL). All recanalization rates after PTPE were 17.2%. However, all cases had recanalization beyond the third portal branches. No cases exhibited recanalization at second or first portal branches. Two cases of all 10 recanalization cases needed additional PVE, that is 1 case was performed PTPE, 1 case was performed TIPE. Recanalization cases showed that the mean RLVs before and after PTPE were 304.3 ± 23 mL and 397.8 ± 53.8 mL, respectively (*P* < 0.01). The median IR-RLV was 26.9%. No cases exhibited post-hepatectomy liver failure. Regarding 8 cases could not achieve our criteria for hepatectomy at 2 weeks after PVE, 2 cases achieved our criteria for hepatectomy at 4 weeks after PVE, 3 cases at 5 weeks, 1 case at 6 weeks, 1 case at 7 weeks, and 1 case at 8 weeks.

**Table 2:** Liver volume and resection rates before and after PTPE

	Total liver volume (ml)	Remnant liver volume (ml)	Effective liver resection rate (%)
Before PTPE	1383.4 ± 395.5	438.6 ± 106	63.8 ± 7.4
2 weeks after PTPE	1394.9 ± 459.2	558.8 ± 99.9	54.8 ± 7.3

*PTPE* percutaneous transhepatic portal embolization

### Correlation between IR-RLV and high IR-pFV cases

We evaluated the IR-RLV of the cases with regard to an IR-pFV greater than 100% on days 1, 3, 5, and 7 after PTPE using ROC (receiver operating characteristics) curves to determine the cut-off values for IR-RLV. The values were 29.2% (area under the curve [AUC] = 0.5297, sensitivity = 59.1%, specificity = 57.7%), 37.3% (AUC = 0.6746, sensitivity = 52.4%, specificity = 74.1%), 42.4% (AUC = 0.6974, sensitivity = 47.8%, specificity = 96.0%), and 40.7% (AUC = 0.5661, sensitivity = 42.9%, specificity = 85.0%), respectively.

### Clinicopathological characteristics in the CH and MH groups

The patients were then classified into two groups, the CH and MH groups, based on an IR-RLV cut-off value of 40% from the above results.

The clinicopathological characteristics of the CH and MH groups are provided in Table 3. The following variables were significantly different between these 2 groups: white blood cell count (4966 ± 1198/μL vs 6987 ± 2625/μL; *P* < 0.01), T-bil (0.98 ± 0.53 mg/dL vs 1.59 ± 0.94 mg/dL; *P* = 0.02), ICGR15 (10.6 ± 3.7% vs 16.1 ± 3.9%; *P* < 0.01), PVP after PTPE (18.2 ± 1.9 cmH_2_O vs 20.5 ± 3.7 cmH_2_O; *P* = 0.03), IR-pFV by US on day 3 after PTPE (156 ± 96% vs 89 ± 57%; *P* < 0.01), and pathological liver fibrosis [6.7% (1/15) vs 42.4% (14/33); *P* = 0.01].

**Table 3:** Clinicopathological characteristics in the considerable hypertrophy and minimal hypertrophy groups by univariate analysis

Characteristics	CH (*n* = 15)	MH (*n* = 33)	*P*
Epidemiology			
Age	63 ± 12	69 ± 7	0.10
Sex (male/female)	11/4	24/9	0.96
HBs-Ag positive (%)	1/15 (6.7%)	8/33 (24%)	0.14
HCV-Ab positive (%)	0/15 (0%)	1/33 (3%)	0.49
Biochemical factors			
WBC (/μL)	4966 ± 1198	6987 ± 2625	< 0.01
Hb (g/dL)	12.4 ± 1.5	13.1 ± 1.0	0.07
Plt (× 10^4^/μL)	23.5 ± 7.9	24.1 ± 7.5	0.81
CRP (mg/dL)	0.9 ± 1.3	0.9 ± 1.7	0.98
PT (%)	92.1 ± 27.6	94.5 ± 15.1	0.70
Total bilirubin (mg/dL)	0.98 ± 0.53	1.59 ± 0.94	0.02
AST (IU/L)	53.7 ± 27.5	63.4 ± 57.4	0.53
ALT (IU/L)	67.9 ± 51.4	84.3 ± 83.3	0.48
Che (IU/L)	231.6 ± 53.6	250.6 ± 66.9	0.33
Alb (g/dL)	3.8 ± 0.5	3.9 ± 0.4	0.36
HbA1c (%)	5.6 ± 0.9	5.5 ± 0.9	0.68
ICGR15 (%)	10.6 ± 3.7	16.1 ± 3.9	< 0.01
99mTc-GSA scintigraphy			
LHL15	0.914 ± 0.029	0.915 ± 0.074	0.95
Portal pressure			
PVP (cmH_2_O) before PTPE	13.2 ± 2.3	14.1 ± 3.3	0.34
PVP (cmH_2_O) after PTPE	18.2 ± 1.9	20.5 ± 3.7	0.03
Portal flow volume			
IR-pFV (%) on day 1	112 ± 90	90 ± 81	0.40
IR-pFV (%) on day 3	155 ± 96	90 ± 59	< 0.01
IR-pFV (%) on day 5	150 ± 108	111 ± 96	0.22
IR-pFV (%) on day 7	160 ± 112	109 ± 95	0.11
Histological factors			
Fibrosis (f2,3,4) (%)	1/15 (6.7%)	14/33 (42.4%)	0.01

*HBs-Ag* hepatitis B surface antigen, *HCV-Ab* hepatitis C virus antibody, *WBC* white blood cell, *Hb* hemoglobin, *Plt* platelet, *CRP* C-reactive protein, *PT* prothrombin time, *AST* aspartate aminotransferase, *ALT* alanine aminotransferase, *Che* cholinesterase, *Alb* albumin, *HbA1c* A1c glycated hemoglobin, *ICGR15* indocyanine green retention test at 15 min, *99mTc-GSA* technetium 99 m diethylenetriaminepentaacetic acid-galactosyl-human serum albumin, *LHL15*, receptor index: uptake ratio of the liver to that of the liver plus heart at 15 min of technetium 99 m diethylenetriaminepentaacetic acid-galactosyl-human serum albumin scintigraphy, *PVP* portal venous pressure, *PTPE* percutaneous transhepatic portal embolization, *IR-pFV* increase rate of portal venous flow volume

**Fig. 1:**
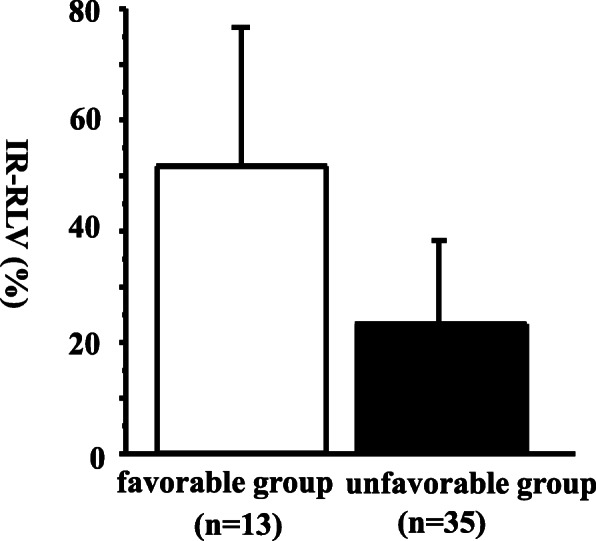
IR-RLV according to the presence of favorable conditions, namely an IR-pFV on day 3 ≥ 100% and an ICGR15 ≤ 15%. Patients with an IR-pFV on 3 days after PTPE ≥100% and an ICGR15 ≤ 15% (favorable group) exhibited a significantly increased IR-RLV compared with other patients (unfavorable group) (*P* < 0.01)

Multivariate analysis with logistic regression revealed that ICGR15 (odds ratio 0.5836, 95% confidence interval: 0.3432–0.9922, *P* = 0.04) and IR-pFV by US on day 3 after PTPE (odds ratio 1.0257, 95% confidence interval: 1.0018–1.0501, *P* = 0.03) were independent favorable factors of increased RLV (Table 4). In addition, patients with an ICGR15 ≤ 15% and an IR-pFV on day 3 after PTPE ≥100% (favorable group, *n* = 13) exhibited significantly increased IR-RLV compared with the other patients (unfavorable group, *n* = 35) (51.6 ± 24.9% vs 23.1 ± 15.1%, *P* < 0.01) (Fig. 1).

**Table 4:** Factors indicative of considerable hypertrophy

Factors	Odds ratio	95% CI	*P*
WBC	0.9989	0.9977–1.0001	0.07
Total bilirubin	1.8378	0.3662–9.2247	0.45
ICGR15	0.5836	0.3432–0.9922	0.04
Fibrosis (f2,3,4)	0.1305	0.0036–4.6704	0.26
PVP after PTPE	0.7307	0.4616–1.1568	0.18
IR-pFV on day3	1.0257	1.0018–1.0501	0.03

*WBC* white blood cell, *ICGR15* indocyanine green retention test at 15 min, *PVP* portal venous pressure, *PTPE* percutaneous transhepatic portal embolization, *IR-pFV* increase rate of portal venous flow volume

Table 5 showed multivariate analysis for factors turned out before PTPE.

**Table 5:** Factors known before PTPE indicative of considerable hypertrophy

Factors	Odds ratio	95% CI	*P*
WBC	0.9994	0.9988–1.0001	0.07
Total bilirubin	0.7503	0.1657–3.3977	0.70
ICGR15	0.6514	0.4736–0.8959	< 0.01

*WBC* white blood cell, *ICGR15* indocyanine green retention test at 15 min

Correlation between the IR-pFV at the non-embolized lobe by US and IR-RLV.

**Fig. 2:**
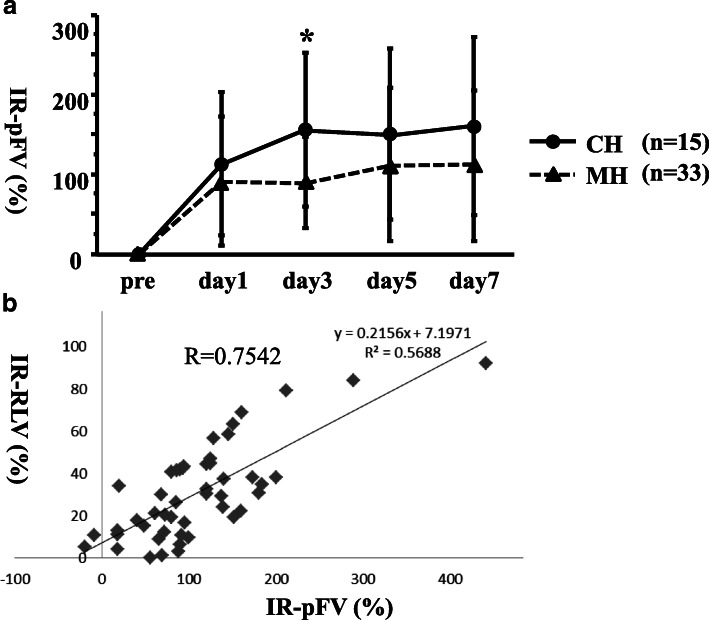
**a** IR-pFV by US after PTPE. In the CH group, the IR-pFV continued to increase through day 1 until day 3. After day 3, the IR-pFV generally remained unchanged until day 7. The IR-pFV generally remained unchanged after day 1 in the MH group**.** *P < 0.01. **b** Correlation between the IR-pFV at the non-embolized lobe by US on day 3 and the IR-RLV. The correlation coefficient was 0.7542

In the CH group, the IR-pFV continued to increase on days 1 and 3 (113 ± 90% and 156 ± 96%, respectively). After day 3, the IR-pFV was largely maintained (151 ± 108% on day 5 and 160 ± 112% on day 7). Conversely, in the MH group, the IR-pFV generally remained unchanged after day 1 (91 ± 82% on day 1, 89 ± 57% on day 3, 112 ± 97% on day 5, and 110 ± 95% on day 7) (Fig. 2a). The correlation coefficient between the IR-pFV at the non-embolized lobe by US and the IR-RLV on day 3 was 0.7542 (Fig. 2b, Table 6). The correlation coefficients on days 1, 5, and 7 were 0.4613, 0.6272, and 0.5735, respectively (Table 6).

**Table 6:** Correlation between the IR-pFV at the non-embolized lobe by US and the IR-RLV

Days after PTPE	Correlation coefficients
Day 1	0.4613
Day 3	0.7542
Day 5	0.6272
Day 7	0.5735

*PTPE* percutaneous transhepatic portal embolization, *IR-pFV* increase rate of portal venous flow volume, *IR-RLV* increase rate of remnant liver volume, *US* ultrasound sonography

## Discussion

We investigated the favorable factors for remnant liver hypertrophy and hemodynamics of portal venous flow volume after PTPE. Univariate analysis revealed that the white blood cell count, T-bil, ICGR15, PVP after PTPE, IR-pFV at the non-embolized lobe by US on day 3 after PTPE and pathological liver fibrosis were significant favorable factors for hepatic hypertrophy after PTPE. Multivariate analyses indicated that the ICGR15 and IR-pFV at the non-embolized lobe by US on day 3 after PTPE were independent favorable factors for hepatic hypertrophy after PTPE. We observed a strong positive correlation between the IR-pFV at the non-embolized lobe by US on day 3 after PTPE and the IR-RLV at 2 weeks after PTPE. In addition, we also demonstrated that the pFV after PTPE stabilized from day 3 after PTPE, even in a group of patients exhibiting increased hypertrophy. In this study, patients with an ICGR15 ≤ 15% and IR-pFV on day 3 after PTPE ≥100% were more likely to exhibit extremely favorable hepatic hypertrophy.

Rous et al. [10] found that ligation of a branch of the portal vein in rabbits resulted in marked atrophy of the parenchyma of the corresponding hepatic lobe with the lobe with uninterrupted portal flow exhibiting regenerative hypertrophy. Subsequently, Makuuchi et al. [11] stated that PVE increased the safety of major hepatectomy for hilar bile duct carcinoma. Kinoshita et al. [12] reported the utility of preoperative PVE before hepatectomy for HCC. Currently, preoperative PVE is widely used for safely performing major hepatectomy for these hepatobiliary malignant tumors [3]. PVE is classified as transileocolic portal embolization (TIPE) or PTPE according to the specific approach used. Whereas TIPE is performed via laparotomy under general anesthesia, PTPE is performed using a puncture technique with ultrasonic guidance under local anesthesia. Therefore, PTPE is more convenient and is currently more commonly used.

Some reports have demonstrated that the absolute increase in the hypertrophy of the future remnant liver ranged from 28.8 to 43% [13, 14]. In this study, the value was 30.5% and thus consistent with previous reports. Regarding procedure-related complications, previous reports reported rates of 12.8 to 14.9% [15, 16]; the rate in our study was 1.7%.

Mars et al. [17] reported that urokinase-type plasminogen activator was immediately increased after partial hepatectomy. Urokinase-type plasminogen activator is induced by shear stress caused by increased blood flow [18] and activates hepatocyte growth factor [19]. Furthermore, nitric oxide (NO) is also induced by shear stress [20]. NO is produced by cytokine-inducible NO synthase (iNOS) and endothelial NO synthase (eNOS). Regeneration was inhibited after partial hepatectomy in mice with iNOS or eNOS inhibition [21, 22]. Therefore, shear stress and blood flow might influence hepatic regeneration.

Recently, it was reported that the mechanisms of hepatic hypertrophy differ between post-PTPE and post-hepatectomy [23, 24]. Some favorable and unfavorable factors have been identified for hepatic hypertrophy after PTPE, such as age, sex, body mass index, nutrition status, previous chemotherapy, and diabetes mellitus [14, 25]. Chronic liver disease and cirrhosis are unfavorable factors for hypertrophy [3]. In addition, the presence of major portal hypertension and portosystemic shunts are unfavorable factors for hypertrophy [3, 26]. Our univariate analysis also demonstrated that PVP after PTPE and pathological liver fibrosis were significant favorable factors for hepatic hypertrophy after PTPE. In general, portal pressure will increase after PVE. Chen et al. [27] reported that patients with PVP below 16 cmH_2_O had a lower incidence of PHLF and patients with PVP of 20 cmH_2_O or above showed lower incidence of grade B or C PHLF. It was reported that the 90-day mortality was associated with PVP greater than 21 mmHg by Allard et al. [28]. Modulation of PVP may be important for better outcome.

Mihara et al. [29] reported that the ICG plasma clearance rate was a significant predictive factor for hypertrophy after PTPE. Kageyama et al. [30] reported that ICGR15 > 20% and T-bil > 1.5 mg/dL were unfavorable factors for hypertrophy after PTPE. These previous findings are consistent with our results.

The frequency of recanalization after PTPE ranges between 7.1 and 33% [31, 32]. In this study, the recanalization rate was 17.2%. However, no cases exhibited recanalization at second or first portal branches. Some reports demonstrate that recanalization is a significant risk factor for poor hypertrophy [32, 33] possibly because pFV might be reduced at the non-embolized lobe in recanalized patients. Thus, we excluded recanalized patients.

Goto et al. [34] reported that an increase in the portal blood flow velocity after PTPE on day 1 correlated with the hypertrophy rate. Our univariate and multivariate analyses revealed that the pFV on day 3 highly correlated with increased hypertrophy after PTPE. Regarding the time course, the previous study demonstrated that the increase in the portal blood flow velocity after PTPE peaked on day 1 [34]. In contrast, we found that the increase in the portal blood flow after PTPE peaked on day 3 in the CH group and day 1 in the MH group. This discrepancy might be the key to good hypertrophy. Hayashi et al. [35] reported that the increase in the serum bile acid levels on day 3 after PTPE was a useful predictor of favorable hypertrophy. Using a portal vein ligation model in rats, Takamura et al. [24] demonstrated that the liver regeneration rate peaked between days 2 and 3 in the non-ligated lobe. Thus, we believe that the IR-pFV on day 3 after PTPE might be an important factor of hepatic hypertrophy after PTPE.

The limitation of this study is that the results may not really apply to the common population of PVE (liver metastases) because most patients were cirrhotic patients or cholestatic patients.

## Conclusions

Preserved liver function, including ICGR15, and an increase in portal venous flow on day 3, i.e., portal venous flow shifted and added for the non-embolized lobe, are important for hepatic hypertrophy.

## Abbreviations

AUC: Area under the curve
CH: Considerable hypertrophy
CT: Computed tomography
eNOS: Endothelial NO synthase
ICGR15: Indocyanine green retention test at 15 min
iNOS: Cytokine-inducible NO synthase
IR-pFV: Increase rate of portal venous flow volume
IR-RLV: Increase rate of remnant liver volume
LHL15: Receptor index: uptake ratio of the liver to the liver plus heart at 15 min of technetium 99 m diethylenetriaminepentaacetic acid galactosyl human serum albumin scintigraphy
MH: Minimal hypertrophy
NO: Nitric oxide
pFV: Portal venous flow volume
PHLF: Posthepatectomy liver failure
PTPE: percutaneous transhepatic portal embolization
PVE: Portal vein embolization
PVP: Portal venous pressure
RLV: Remnant liver volume
ROC: Receiver operating characteristics
T-bil: Total bilirubin
TIPE: Transileocolic portal embolization
US: Ultrasound sonography
